# Meristem Initiation and *de novo* Stem Cell Formation

**DOI:** 10.3389/fpls.2022.891228

**Published:** 2022-04-26

**Authors:** Antoine Nicolas, Patrick Laufs

**Affiliations:** ^1^Université Paris-Saclay, INRAE, AgroParisTech, Institut Jean-Pierre Bourgin (IJPB), Versailles, France; ^2^Université Paris-Saclay, Orsay, France

**Keywords:** *Arabidopsis thaliana*, rice, stem cells, *CLAVATA3*, *WUSCHEL*, *HAIRY MERISTEM*, axillary meristem

## Abstract

Plant aerial development relies on meristem activity which ensures main body plant axis development during plant life. While the shoot apical meristem (SAM) formed in the embryo only contributes to the main stem, the branched structure observed in many plants relies on axillary meristems (AMs) formed post-embryonically. These AMs initiate from a few cells of the leaf axil that retain meristematic characteristics, increase in number, and finally organize into a structure similar to the SAM. In this review, we will discuss recent findings on *de novo* establishment of a stem cell population and its regulatory niche, a key step essential for the indeterminate fate of AMs. We stress that *de novo* stem cell formation is a progressive process, which starts with a transient regulatory network promoting stem cell formation and that is different from the one acting in functional meristems. This transient stage can be called premeristems and we discuss whether this concept can be extended to the formation of meristems other than AMs.

## Introduction

Plants are characterized by continuous organogenesis and growth throughout their life by the action of meristems. These structures are formed by a few hundreds or thousands of cells, depending on the species, that are maintained undifferentiated and proliferating by the combined action of meristematic genes such as the KNOTTED1-LIKE Homeobox encoding gene S*HOOT MERISTEMLESS* (*STM*) and an hormonal balance of high cytokinin (CK) to low gibberellin (Shani et al., 2006; Hay and Tsiantis, 2010; Maugarny-Calès and Laufs, 2018; Shi and Vernoux, 2022). Within this population of *STM*-expressing meristematic cells lies a specific subpopulation of semi-permanent stem cells that can be recognized by the expression of the *CLV3* gene (Fletcher et al., 1999). These stem cells divide infrequently to replenish themselves while producing cells contributing to the meristem organogenetic activity. These stem cells are maintained by the activity of a stem cell niche that provides a cellular environment regulating stem cell division and preventing their differentiation (Bäurle and Laux, 2003; Dinneny and Benfey, 2008). While meristem multicellularity and complexity of the regulatory interactions between its different functional domains may be an advantage for the robustness of established meristems, they become, however, challenges to overcome when it comes to producing new meristems. Such new meristems are nevertheless repetitively produced during the plant life, turning for instance into axillary meristems (AMs) that increase the branching pattern of the plant. Here, we will discuss current knowledge and hypothesis of how an organized meristem emerges from a small group of meristematic cells, concentrating mostly on the formation of the stem cell population and its regulatory niche.

### How Are Stem Cells Regulated in an Arabidopsis Meristem?

In the apical part of the shoot apical meristem (SAM) lies a group of semi-permanent stem cells maintained in their undifferentiated and pluripotent state by an underlying organizing center (OC) that contributes to the stem cell niche function (Laux et al., 1996). The *WUSCHEL* (*WUS*) gene is a meristematic stem cell fate regulator expressed in the OC and encoding a HOMEOBOX-like transcription factor that moves through plasmodesmata to promote *CLAVATA3* (*CLV*) expression in the stem cell pool (Laux et al., 1996; Mayer et al., 1998; Yadav et al., 2011; Daum et al., 2014). To ensure its function in establishing the stem cell niche, WUS proteins form homodimers but also act through monomers or heterodimers with STM (Perales et al., 2016; Rodriguez et al., 2016; Sloan et al., 2020; Su et al., 2020). In turn, the small secreted peptide CLV3 inhibits *WUS* expression upon binding to receptor kinases such as *CLV1*, *CLV2*, *CORYNE* (*CRN*), or *BARELY ANY MERISTEM 1–3* (*BAM1-3*; Clark, 2001; Brand et al., 2002; DeYoung et al., 2006; DeYoung and Clark, 2008; Müller et al., 2008; Ogawa et al., 2008; Schlegel et al., 2021). This core regulatory network contributes to the maintenance of the stem cell population while additional interacting regulators such as the HAIRY MERISTEM (HAM) transcription factors contribute to the positioning of the stem cells. WUS can bind and form heterodimers with HAM proteins to define the expression domain of *CLV3* (Zhou et al., 2015, 2018). *HAM1* and *HAM2* are expressed in domains partially overlapping *WUS* domains. Specifically, both genes are expressed in the medullar and peripheral zones of the apical meristem but not in the L1 or L2 layers of the central zone (Schulze et al., 2010; Zhou et al., 2018; Han et al., 2020a). Expression and modeling data suggest that an apical-basal gradient of *HAM1/2* genes is established in the apical meristem to define *CLV3* expression pattern in the SAM. The establishment of the *HAM* gradient is in part mediated by miR171 that target the *HAM* genes and the transcription factor ARABIDOPSIS THALIANA MERISTEM LAYER 1 (ATML1) that promotes miR171 expression in the epidermis (Wang et al., 2010; Takanashi et al., 2018; Han et al., 2020b). Furthermore, an epidermal signal of CK is associated with the *CLV-WUS* genetic network. Indeed, modeling suggests that a combination of long and short range epidermal signals that could be CK production and response, respectively, act as positional cues for patterning the *WUS* domain (Leibfried et al., 2005; Chickarmane et al., 2012; Gruel et al., 2016). Finally, auxin signaling is locally controlled to maintain a low level of auxin response in the stem cell niche because the presence of a high level of auxin signaling could induce organ emergence from the center of the meristem and severely disturb meristem integrity (Shi et al., 2018; Ma et al., 2019; Galvan-Ampudia et al., 2020). Altogether, this network contributes to maintenance of stem cell homeostasis via a balance between their loss and renewal and proper spatial positioning of the stem cell and stem cell niche to allow meristem activity to respond to environmental signals (Yoshida et al., 2011; Pfeiffer et al., 2016; Landrein et al., 2018).

### How Is an Arabidopsis Axillary Meristem Initiated?

**Figure 1:**
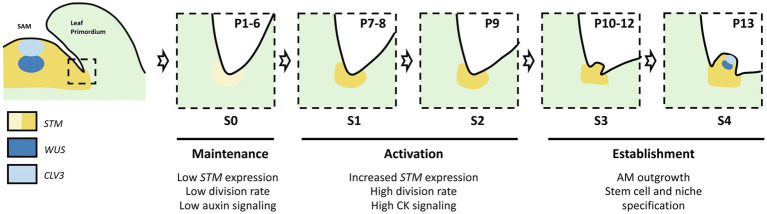
Steps of axillary meristem formation in the axils of *Arabidopsis thaliana* rosette leaves. Developmental stages (S0 to S4) are shown relative to leaf age (defined in plastochrones, P1 to P13).

The initiation of an AM during the vegetative phase in the axils of the rosette leaves of *Arabidopsis* has been extensively studied and several stages have been defined based on cellular and morphological features (Long and Barton, 2000; Xin et al., 2017; Figure 1). Thus, axils of leaf primordia 1–6 (P1-6, counted from the SAM) show no morphological or cellular evidence of AM initiation (stage S0). At S1, P7–8 leaf axils show cell divisions which intensify at S2 in P9 axils. Then, at S3, a bulge forms in P10-12 axils and becomes a dome-shaped structure at S4 in P13 axils, and primordia start to emerge from the S5 AM at ≥ P14.

Thus, AM initiation can be divided into two phase, a maintenance phase during which some meristematic cells remain latent in the leaf axils (stage S0) and an activation phase in which the division of these cells leads to the formation of an AM (S1 to S4; Grbić and Bleecker, 2000; Long and Barton, 2000). Live imaging showed that there are only a very limited number of cell divisions in the latent axillary meristem, which contributes to limiting the risk of somatic mutations that could be propagated in the axillary branches (Burian et al., 2016). During the maintenance phase, *STM* remains expressed at a low level in the leaf axil and keeps cells in an undifferentiated state in contrast to their neighboring cells which undergo differentiation. Such maintenance of *STM* expression requires depletion of auxin from the axillary region and involves at the molecular level a self-activation loop facilitated by a permissive epigenetic environment (Wang et al., 2014a,b; Cao et al., 2020). During the activation phase, *STM* expression increases to induce division of these cells and the bulging out of the initiating AM (Shi et al., 2016; Xin et al., 2017). The importance of *STM* in AM formation is illustrated by the absence of AM formation in a high proportion of leaf axils in the weak allele of *STM*, *stm-bumpershoot1* (Shi et al., 2016). A local pulse of CK response is required to stimulate *STM* expression and promote AM formation, possibly through a mutual positive feedback loop between *STM* and CK (Wang et al., 2014b). Multiple factors, such as *CUP-SHAPED COTYLEDON 1–3* (*CUC1-3), LATERAL SUPPRESSOR* (*LAS*), *REVOLUTA* (*REV)*, *DORNRÖSCHEN* (*DRN*) and *DORNRÖSCHEN LIKE* (*DRNL*), *REGULATOR OF AXILLARY MERISTEMS 1–3* (*RAX1-*3), *REGULATOR OF AXILLARY MERISTEM FORMATION* (*ROX*), and *ARGONAUTE 10* (*AGO10*), provide spatial and temporal cues for the local activation of *STM* expression, and thus control the pattern of AM formation (Greb et al., 2003; Hibara et al., 2006; Keller et al., 2006; Müller et al., 2006; Raman et al., 2008; Yang et al., 2012; Shi et al., 2016; Zhang et al., 2018, 2020).

### How Is a New Stem Cell Niche Formed in an Arabidopsis Axillary Meristem?

**Figure 2:**
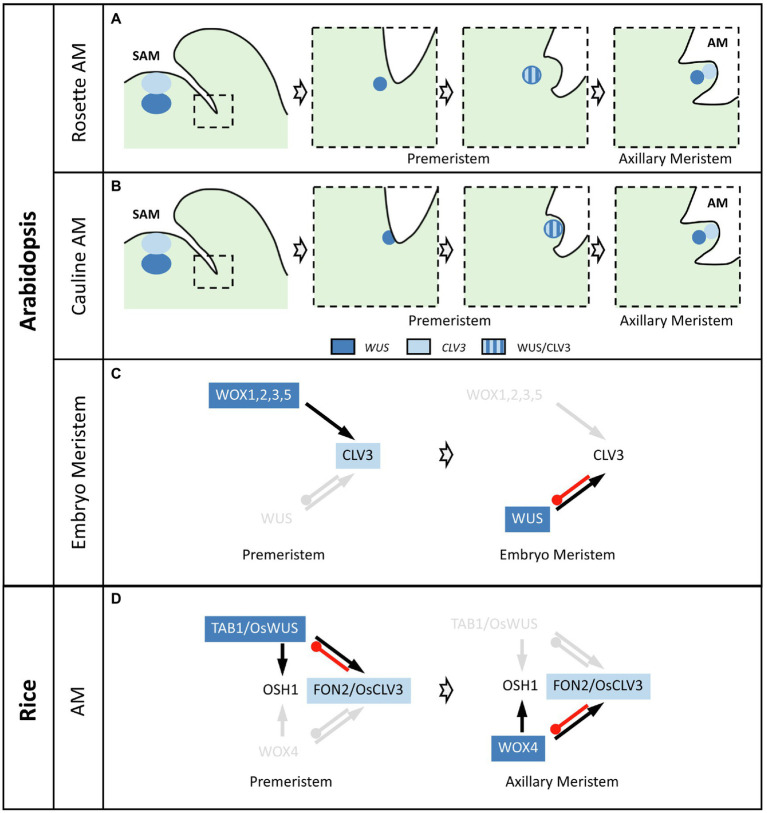
Regulatory dynamics driving *de novo* stem cell formation during meristem formation. **(A)** Stem cell formation in *Arabidopsis thaliana* rosette AMs. Neither *WUS* nor *CLV3* are expressed in the axils of young rosette leaf primordia from which the AM will initiate (first panel). *WUS* expression becomes expressed in the inner part of the axil (second panel) and later activates *CLV3* in an inner, overlapping domain (third panel). These stages can be defined as premeristem. Finally, *WUS* and *CLV3* expressions resolve in two separate domains, in the inner and apical part of the rosette AM, respectively (last panel). **(B)** Stem cell formation in *Arabidopsis thaliana* cauline AMs. Neither *WUS* nor *CLV3* are expressed in the axils of young cauline leaf primordia from which the AM will initiate (first panel). *WUS* expression becomes expressed in the apical part of the axil (second panel) and later activates *CLV3* in an apical, overlapping domain (third panel). These stages can be defined as premeristem. Finally, *WUS* and *CLV3* expressions resolve in two separate domains, in the inner and apical part of the cauline AM, respectively (last panel). **(C)** Stem cell formation in *Arabidopsis thaliana* embryo meristems*. CLV3* expression is activated by the *WOX1*, *2*, *3*, and *5* genes at the premeristem stage, while in established and active meristems *WUS* activates *CLV3* expression. **(D)** Stem cell formation in rice AMs. *FON2* (the *CLV3* ortholog) expression is activated by *TAB1* (the *WUS* ortholog) at the premeristem stage, while in established and active meristems *WOX4* activates *FON2* expression. *TAB1* or *WOX4* promotes *OSH1* (the *STM* ortholog) expression in premeristems and meristems, respectively. In **(C)** and **(D)**, black arrows mean expression activation while red lines mean repression. Genes and interactions indicated in light gray are not present at the described stage.

The observation that AMs result from the division of a few cells maintaining *STM* expression in the axils is in agreement with the so-called “detached meristem” concept for AM formation (Steeves and Sussex, 1989). In this “detached meristem” model, meristematic cells “detached” from the SAM are maintained in the axil of the leaf before being amplified to generate the new AM (Long and Barton, 2000). This model is opposed to the “*de novo* origin of AM” model, in which differentiated cells regain a meristematic fate to form a new AM (McConnell and Barton, 1998). However, while cells of the “detached meristem” express meristem markers such as *STM*, they express neither the stem cell marker *CLV3* nor *WUS*, clearly indicating that they are not yet organized in a properly structured meristem (Figure 2A). In fact, *WUS* becomes activated only at S1 after the increase in *STM* expression and WUS activates *CLV3* expression at S2 (Wang et al., 2017; Xin et al., 2017). A similar temporal succession of *WUS* and *CLV3* activation is also observed during AM establishment in cauline leaf axils of *Arabidopsis* (Nicolas et al., 2022). Interestingly, in both rosette and cauline AM, *WUS* and *CLV3* are initially expressed in overlapping domains and not in mostly separated domains as in the SAM. More precisely, in cauline AMs, *WUS* and *CLV3* are initially expressed in overlapping apical domains while later *WUS* expression shifts down from the apical to a central domain (Nicolas et al., 2022; Figure 2B). The opposite dynamic is observed in rosette AMs, as *WUS* and *CLV3* are co-expressed in the center of S4 rosette AM while later on, *CLV3* expression domain shifts upwards and relocates to an apical position (Figure 2A; Xin et al., 2017). This spatial rearrangement of *CLV3* expression in rosette AMs is mainly ensured by the establishment of an apico-basal gradient of *HAM* gene expression. The role of the *HAM* genes during AM formation is demonstrated by the phenotype of the triple mutant *ham1 ham2 ham3* in which *CLV3* expression does not shift from a central to an apical domain and AM formation is compromised. Complementation of the triple *ham1 ham2 ham3* mutant with each of the *HAM* genes showed that *HAM1* and *2* play the most prominent role for *CLV3* expression pattern dynamics during AM formation. Interestingly, the apico-basal gradient of *HAM* gene expression is established progressively during AM formation. Indeed, the *HAM* genes are first expressed uniformly at S2 and S3 and it is only from S4 and S5 onwards that a gradient of expression is formed. Such a gradient is in part a consequence of the epidermis-specific expression of their negative regulators miR171 (Zhou et al., 2018; Han et al., 2020a,b).

What drives the formation of a new stem cell population and its niche is not fully understood yet. *WUS* is a central actor in the process as the *wus* mutant has a very strong AM initiation defect in contrast to the *clv3-2* mutant which has only very weak AM initiation defects (Wang et al., 2017; Xin et al., 2017). *De novo WUS* expression in AMs is promoted by CK, through binding of the type-B Arabidopsis response regulator proteins (ARRs), which mediate the transcriptional response to CK, to the *WUS* promoter (Wang et al., 2017). Thus, CK signaling could be a link between the AM activation phase where it forms a positive feedback loop with *STM* and the AM establishment phase where it promotes *WUS* expression. Another link between the early phases of meristem initiation and its establishment is provided by the *CUC* genes. As described before, *CUC* genes are required for AM initiation (Hibara et al., 2006; Raman et al., 2008), likely by preventing cell differentiation and maintaining cells in a meristematic fate. However, recent results show that expression of those boundary genes has to be downregulated from the initiating meristem to proceed to the establishment phase and allow stem cell formation (Nicolas et al., 2022). *CUC* ectopic expression in the developing AM perturbs its growth and prevents stem cell establishment as shown by delayed expression of *WUS* and *CLV3*. Repression of the *CUC* genes from the initiating meristem results from the redundant action of two NGATHA-LIKE (NGAL) transcription factors, DPA4 and SOD7. Thus, the boundary identity needs to be repressed in order to allow *de novo* establishment of stem cells in newly formed AM and permit proper expression of *WUS* and *CLV3* (Nicolas et al., 2022).

Altogether, Arabidopsis AM establishment appears as being a gradual process. Although AMs derive from boundary cells that maintain a meristematic fate, a prerequisite for AM establishment is the repression of boundary fate from the developing AM. CK initiates *de novo* stem cell niche establishment by activating *WUS* expression. In parallel, an apico-basal gradient of HAM activity is established which contributes to the dynamic (re)positioning of the stem cells and stem cell niche and the formation of an active meristem.

### How Is a New Stem Cell Niche Formed in a Rice Axillary Meristem?

Development of the rice AMs during the vegetative phase leading to rice tillering provides another example of a dynamic reorganization of the regulatory network leading to *de novo* stem cell niche establishment. In rice, the axillary bud is formed on the side of the main stem (also called culm in rice) and is composed by a protective, modified leaf, the prophyll, enclosing a few leaf primordia and the AM. The first sign of AM initiation is a small group of small, dense cells that readably forms a bulge growing out from the stem in P3 axils, a transient state called “premeristem” by Tanaka et al. (2015). Cells of the premeristem are expressing *OSH1*, a marker for meristematic cells and orthologue of Arabidopsis *STM* (Oikawa and Kyozuka, 2009; Tanaka et al., 2015). In P4, the developing AM grows through cell division forming a cone-like structure initiating first the prophyll and later true leaf primordia. LAX PANICLE1 (LAX1, orthologous to the bHLH transcription factor ROX of *Arabidopsis thaliana*), LAX PANICLE2 (a nuclear protein interacting with LAX1), and MONOCULM1 (orthologous to the GRAS transcription factor LATERAL SUPPRESSOR of *Arabidopsis thaliana*) synergistically promote *OSH1* expression and are required for AM formation (Oikawa and Kyozuka, 2009; Tabuchi et al., 2011).

Apparition of the stem cells in rice AM can be followed by the expression of *FLORAL ORGAN NUMBER2* (*FON2*), that like its Arabidopsis orthologue *CLV3* marks the stem cells in meristems (Suzaki et al., 2006). This revealed an early specification of the stem cells in rice developing AMs as *FON2* expression is detected in a central, apical subset of the premeristem (Tanaka and Hirano, 2020). *fon2* mutants show larger developing AMs with enlarged *FON2* expressing domains, while conversely in *FON2* overexpressors AM have a reduced size, are often flat, and show reduced *OSH1* expression that is not maintained, suggesting that *FON2* is required for AM maintenance (Tanaka and Hirano, 2020).

*TAB1* (*TILLERS ABSENT 1*, also called *MONOCULM3*), the rice orthologue of the *WUS* gene is required for AM formation in rice (Lu et al., 2015; Tanaka et al., 2015; Shao et al., 2019). *TAB1* is expressed early on in the developing AM, in a central, apical domain of the premeristem that overlaps with the *FON2* expression domain (Tanaka et al., 2015; Tanaka and Hirano, 2020). Like in Arabidopsis, *TAB1* expression is induced by CKs and mutation of *TAB1* leads to the absence of a functional AM, with only the development of the prophyll in some cases (Lu et al., 2015; Tanaka et al., 2015). Accordingly, expression of *OSH1* is strongly reduced in *tab1* mutants, suggesting *TAB1* promotes AM formation through promoting *OSH1* expression to prevent cell differentiation (Figure 2D). In contrast, expression of *LAX1* and *MOC1* is not modified in *tab1* mutants, suggesting that these genes act upstream or in parallel to *TAB1*. However, in contrast to what occurs in Arabidopsis, *TAB1* expression is not maintained in rice AM once the prophyll is initiated (Tanaka et al., 2015). Instead, *WOX4*, the closest *TAB1* paralog becomes expressed in the AM. AM defects of the *tab1* mutant can be partially rescued by expressing *WOX4* under the control of the *TAB1* promoter, showing a strong functional conservation between WOX4 and TAB1 proteins (Tanaka and Hirano, 2020). Furthermore, *tab1* AM defects are also partially rescued in the *tab1 fon2* double mutant, in which a precocious expression of *WOX4* is observed (Tanaka and Hirano, 2020). Hence, while *TAB1* is required for the formation of the premeristem, *WOX4* is required for the later function of the established AM, which is in agreement with *WOX4* being required for SAM maintenance during rice vegetative development and *TAB1* being not required (Ohmori et al., 2013; Tanaka et al., 2015).

Altogether these observations indicate that AM formation in rice involves a transient phase, called premeristem, and that the stem cell promoting role of *WUS* during the premeristem stage is later taken over by *WOX4* in functional meristems (Figure 2D).

### Can the Concept of Premeristem Be Extended to All Axillary Meristems?

The concept of premeristem was coined by Tanaka et al. (2015) to describe a transient stage when rice AMs are formed by a group of cells with meristematic features but are not yet alike functional meristems. In rice, a difference between axillary premeristem and established AM is the involvement of the *WUS* ortholog *TAB1* instead of *WOX4*. In contrast, in the case of Arabidopsis, expression and genetic data indicate that the same gene, *WUS*, is acting in establishing and active AMs. However, the expression patterns of *WUS* or *CLV3* are reorganized during Arabidopsis AM formation, possibly as a result of *HAM* gene dynamics. We therefore suggest to extend the concept of premeristems to all developing AM that are formed by a group of meristematic cells and in which the regulatory networks are not yet similar to the ones acting in established meristems either because the actors are different as in rice or because the actors are expressed in different domains, as in Arabidopsis. Whether this concept may apply to developing AMs in other species awaits precise molecular and genetic deciphering of the processes at play.

### Can the Concept of Premeristem Be Extended to All Newly Formed Meristems?

Beside AMs, plants form new meristems in other contexts. Depending on the species, floral meristems (FM) have different origins and they initiate a new stem cell population, which is not permanently maintained in relation with the determinate fate of flowers. In the case of Arabidopsis, FMs arise at the flank of the inflorescence SAM. In fact, Arabidopsis FMs are thought as modified AMs, with the subtending leaf being reduced to a cryptic bract which growth can be derepressed in some mutant backgrounds (Long and Barton, 2000; Ohno et al., 2004). *WUS* expression starts in FM at late stage 1 and becomes stronger at stage 2 (Mayer et al., 1998; Grégoire et al., 2018; Nicolas et al., 2022) when it induces *CLV3* expression, which thus begins to be expressed at stage 2 (Seeliger et al., 2016; Prunet et al., 2017; Nicolas et al., 2022). Like in the AMs, increased CK signaling is observed in young FMs (Yoshida et al., 2011) and could contribute to *WUS* activation. More recently, it has been shown that the meristem patterning gene *REVOLUTA* together with the main floral determinant *LEAFY,* in part through its target *RAX1,* contribute to *WUS* activation in a partially redundant manner (Denay et al., 2018). Finally, as in AMs, *DPA4* and *SOD7* are required for efficient *de novo* stem cell formation in FMs (Nicolas et al., 2022). Despite these findings, our understanding of the mechanisms at play during stem cell formation in FM lacks the detail required to unambiguously recognize or exclude a transient stage as defined for the premeristem.

The first meristem formed is the embryonic meristem. In embryos, *WUS* is expressed early from the 16-cell stage onwards (Mayer et al., 1998). However, it is not required for the induction of *CLV3*, which starts to be expressed from the transition stage and at the heart stage in the subepidermal layer of the meristem, then extends into the epidermis leading to the formation of the apical meristem (Zhang et al., 2017b). Instead of *WUS,* other genes from the same family, the *WUSCHEL-LIKE HOMEBOX 1* (*WOX1*), *WOX2*, *WOX3*, and *WOX5* genes, are required for stem cell establishment *via* activation and spatial rearrangement of *CLV3* expression (Zhang et al., 2017b). Therefore, formation of the embryonic meristem involves a transient stage during which both the actors (*WOX1,2,3,5* instead of *WUS*) and the expression pattern of the actors (*CLV3* starting with an expression in the inner layers) are different from what is observed in the established SAM (Figure 2C). This transient phase during which *WOX* genes activate *CLV3* expression could be seen as a premeristem stage.

Finally, new meristems can also be formed during *in vitro* culture through different ways (Ikeuchi et al., 2019). In the most widely used protocol, tissue explants are first cultured on an auxin-rich callus-inducing medium and then switched to a shoot-inducing medium characterized by a high CK level that promotes the formation of shoot meristems. CK-mediated activation of *WUS* expression by ARR proteins is a key step of shoot meristem formation, as it can be by-passed by forced *WUS* expression (Meng et al., 2017; Zhang et al., 2017a). Indeed, *WUS* activation results from two successive steps: first, cell divisions erase repressive epigenetic marks on the *WUS* locus, and, second, ARR proteins interact with the class III homeodomain-leucine zipper (HD-ZIP III) transcription factors on the *WUS* locus to promote its expression. This interaction between ARRs and HD-ZIP III allows limiting the expression pattern of *WUS*, as CK-mediated ARR activity is widely present in the regenerating callus while *HD-ZIP III* gene expression is more localized (Zhang et al., 2017a). However, initial expression pattern of *WUS* is reorganized during regeneration to reach the pattern typical of the OC in regenerated meristems (Meng et al., 2017; Zhang et al., 2017a). Shoot meristems can also be formed by direct conversion of lateral root meristems without the formation of an intermediate callus (Rosspopoff et al., 2017). Here again, formation of shoot meristems goes along with the induction of *WUS* expression as a response to CK treatments. Interestingly, during their activation phase, *WUS* and *CLV3* show largely overlapping expression domains that only later resolve into patterns characteristic of *bona fide* meristem (Rosspopoff et al., 2017).

## Conclusion and Perspectives

Formation of new meristems occurs repeatedly during plant development, starting from the embryonic meristem to AMs and FMs. Such structures are formed by meristematic cells that contain at their center a group of stem cells and stem cell niche. To form them, one could envisage two scenarios: the formation of a group of meristematic cells in a first step and in a second step, the definition of a functional stem cells and niche or, alternatively, a progressive increase of the pool of meristematic cells with the successive definition of stem cells niche and stem cells in a dynamic way. The observations discussed above all plead for the second scenario and reveal a transient, dynamic phase which can be called premeristem leading to the formation of functional meristem. Notably, the formation of this premeristem is marked by a transient regulatory network that leads to the regulatory network active in established AM but differs from it, either by identity or by the patterns of the actors involved. Furthermore, some factors such as the *CUC* or *RAX1* genes or CK may contribute to the coordination between the increase of the meristematic cells and the formation of a stem cell niche. In addition, the role of other factors such as the oxidative status or mechanical signals that have been recently shown to regulate the stem cell or its niche could be investigated for their role during meristem formation (Weits et al., 2019; Bhattacharya et al., 2022).

## Author Contributions

AN and PL wrote the review. All authors contributed to the article and approved the submitted version.

## Funding

The IJPB benefits from the support of Saclay Plant Sciences-SPS (ANR-17-EUR-0007).

## Conflict of Interest

The authors declare that the research was conducted in the absence of any commercial or financial relationships that could be construed as a potential conflict of interest.

## Publisher’s Note

All claims expressed in this article are solely those of the authors and do not necessarily represent those of their affiliated organizations, or those of the publisher, the editors and the reviewers. Any product that may be evaluated in this article, or claim that may be made by its manufacturer, is not guaranteed or endorsed by the publisher.
